# Body Fat Mass and Risk of Cerebrovascular Lesions: The PRESENT (Prevention of Stroke and Dementia) Project

**DOI:** 10.3390/ijerph16162840

**Published:** 2019-08-08

**Authors:** Im-Seok Koh, Yang-Ki Minn, Seung-Han Suk

**Affiliations:** 1 Department of Neurology, National medical center, Seoul 04564, Korea; 2 Department of Neurology, Kangnam Sacred Hospital, Hallym University, Seoul 07441, Korea; 3 Department of Neurology, Wonkwang University, Sanbon Medical Center, Gunpo 15865, Korea

**Keywords:** obesity, body mass index, ischemic stroke, risk factor, absolute fat mass

## Abstract

Obesity is known to increase the risk of stroke. It is unclear whether high absolute fat mass (FM) increases the risk of stroke independently. We studied the correlation between FM and silent brain infarction/white matter change (SI/WMC) using brain computed tomography. We selected subjects from the local government health promotion project. We randomly selected a target population that had never been diagnosed with stroke or dementia. FM was measured by bioelectrical impedance analysis (BIA). We divided the subjects into three groups according to the FM (gender-specific tertiles [GTx]). Seven hundred and twenty-two subjects (321 men) between 50 and 75 years of age were recruited. The overall odds ratio (OR) of SI/WMC was 2.23 (95% confidence interval (CI), 1.34–3.71; *p* = 0.002) times higher in the 37th to 100th percentiles (GT_3_) than in the first to 32nd percentiles (GT_1_). When men and women were separated, the OR of GT_3_ was 1.35 (CI, 0.62–2.94; *p* = 0.45) in men and 3.2 (CI, 1.60–6.40; *p* = 0.001) in women. The findings were not found to be statistically significant after adjusting for the well-known stroke risk factors. When the subjects were divided into a high FM (HFMG, GT_3_) and low FM group (LFMG, GT_1_ + GT_2_), the HFMG showed an increased OR of SI/WMC in women. Similar results were seen after adjusted (overall: OR, 1.38; CI, 0.85–2.25, *p* = 0.198; men: OR, 0.93; CI, 0.422–2.051; *p* = 0.86; women: OR, 2.02; CI, 1.06–3.86; *p* = 0.03). The findings suggest that high FM may be an independent risk factor for ischemic stroke among adults free from stroke and dementia, especially in women.

## 1. Introduction

Obesity reflects an excess of body fat. Consequently, obesity has been recognized as an important cause of cerebral infarction [1]. However, obesity is often associated with other well-known risk factors of cerebral infarction, such as hypertension, diabetes, and hyperlipidemia. Hence, it is unclear whether body fat itself is a true risk factor for cerebral infarction [1]. It has recently been reported that obesity causes chronic inflammatory reactions via adipocytes and thus causes atherosclerosis [2,3]. 

Obesity is usually defined using the body mass index (BMI), following a 19th century report by Quetlet that body weight varies across adults with the square of height [4]. BMI is a simple index of weight-for-height that is commonly used to classify adults as underweight, overweight, or obese. It is defined as the weight in kilograms divided by the square of the height in meters (kg/m^2^). An adult with a BMI of ≥30 kg/m^2^ is generally classified as obese [5,6].

Because height and weight can be easily measured, BMI could be a good indicator of cerebral infarction in cases where obesity is indirectly responsible through associated hypertension and diabetes. However, given that BMI does not distinguish between weight associated with muscle and that with fat [7,8,9], it is not an accurate indicator for chronic inflammation and cerebral infarction caused by elevated amounts of fat. 

After adjusting for other cardiovascular risk factors, BMI was shown to not be a high-level risk factor for stroke [5,6]. Some studies have reported a complete lack of correlation between BMI and stroke [10,11,12]. Several large-scale studies have suggested that abdominal obesity, rather than BMI or general obesity, is more closely related to stroke risk, especially in men [13,14,15].

If obesity (high BMI) is not a causative factor for stroke but the presence of excess fat cells are, then the total fat mass (FM) would be a better indicator of cerebral infarction risk than BMI. However traditional methods for the measurement of FM such as dual-energy X-ray absorptiometry are difficult, expensive, and use ionized. Recently, the bioelectrical impedance analysis (BIA) method was developed to measure total FM directly [16]. 

We hypothesized that absolute FM, rather than BMI, is a risk factor for cerebral infarction. The purpose of this study was to establish a link between total FM and ischemic stroke using BIA.

## 2. Materials and Methods 

### 2.1. Subject Recruitment

We recruited subjects from health promotion projects conducted by the local government (Prevention of Stroke and Dementia (PRESENT) project). The PRESENT project was started in July 2007, supported by the regional government in Ansan City, Gyeonggi-do, Korea [17,18]. The goal of the PRESENT project was to prevent dementia and cerebral infarction through early medical check-ups [18].

As a part of the PRESENT project, we recruited people between the ages of 50 and 75 years who had never been diagnosed with cerebral infarction or dementia in Ansan City between 2007 and 2009, based on systemic random sampling using a previously described method (the details are described below) [18]. Inclusion criteria were adults 50–75 years of age who are registered as residents of Ansan city, had no history of stroke and dementia, and agreed to a free health check-up at Ansan public health center. Subjects were asked if they had been previously diagnosed with cerebral infarction or dementia in a telephonic interview. The subjects were not provided with specific definitions of cerebral stroke or dementia and we did not distinguish between different types of stroke (ischemic or hemorrhagic) or stages of dementia (dementia or mild cognitive impairment). The study protocol was reviewed and approved by the institutional review board of Wonkwang University Hospital (WKIRB-201611-BM-071). All procedures were performed after written consent was obtained from each subject. 

Subjects were systemically selected using a registered list in the regional government office. The phone numbers provided on the list were used to make telephone calls. 

We contacted every 100th person on the list, with whom a trained nurse conducted a telephonic interview. If a potential participant could not be contacted, refused to participate, had moved, or had a history of stroke or dementia, we contacted the next person on the list [17,18]. The telephonic interview was conducted three times in 2007, 2008, and 2009. A total of 2843 people were selected from the registered list (539 in 2007, 1064 in 2008, and 1240 in 2009) [17,18]. As of 2009, the population of Ansan City belonging to the age group of 50 to 75 years was 131,866. Of the 2843 people, 1119 people could not be contacted, 779 refused to participate, 100 moved out of Ansan City, 49 had a history of stroke or dementia, and 18 died before examination. This left us with 778 subjects (27.4% of the original pool), who underwent examination at a community health center. Thus, a total of 778 subjects were evaluated. Out of the 778 subjects, we excluded those for whom education level data (54 subjects) or BIA data (four subjects) were missing [18]. 

### 2.2. Risk Factor Assessment

Known risk factors for ischemic stroke, such as hypertension, education, smoking, diabetes mellitus, and hypercholesterolemia were evaluated. Hypertension was defined as a previous diagnosis of hypertension, the use of one or more anti-hypertensive drugs, a systolic pressure of ≥140 mmHg, or a diastolic pressure of ≥90 mmHg [19]. Hypercholesterolemia was defined as the use of one or more lipid-lowering agents, fasting total cholesterol levels of ≥240 mg/dL, low-density lipoprotein cholesterol levels of ≥160 mg/dL, or triglyceride levels of ≥200 mg/dL [20]. Diabetes mellitus was defined as a previous diagnosis of diabetes mellitus, the use of anti-diabetic medication (including insulin), or fasting glucose levels of ≥126 mg/dL [21]. Smoking was defined as a current smoking habit [17,18].

### 2.3. Measurement of FM

FM was measured using an InBody720 body composition analyzer (InBody, Seoul, Korea) [14]. The validity of BIA has been documented in previous studies [16,22,23]. Since men were heavier than women, FM was studied separately for men and women. We divided subjects into three groups according to the FM (gender-specific tertiles [GTx]). GT_1_ indicates the first to 32th percentiles, GT_2_ indicates the 33rd to 36th percentiles, and GT_3_ indicates the 37th to 100th percentiles. 

### 2.4. Neuroimaging

The Brilliance^TM^ computed tomography (CT) scanner (six slices; Philips, Eindhoven, The Netherlands) was used for brain CT [17]. Two different neurologists blinded to the subjects’ clinical conditions independently evaluated the results [17]. Subjects were divided into a normal group or a silent infarction/white matter change (SI/WMC) group, according to the brain CT findings. SIs were defined as well-defined areas >2 mm showing attenuation without relevant clinical neurological events [24]. WMCs were defined as ill-defined and moderately hypodense areas of >5 mm located in the periventricular or subcortical area, including extensive periventricular lesions and severe leukoencephalopathy [24,25]. 

### 2.5. Data Analyses

The dependent variable was the presence of SI/WMC on brain CT. Data were analyzed with χ^2^ test, t-test, and multiple regression analysis using the Statistical Package for the Social Sciences (SPSS; Windows version 18, SPSS Inc, Chicago, IL, USA). We obtained odds ratios (ORs) by univariate regression analysis. To obtain adjusted ORs for known stroke risk factors, we used multivariate regression analysis. The independent variables were defined as the three FM GTx. Subsequently, the analysis was conducted by dividing the subjects into two groups, i.e., a high FM group (HFMG, GT_3_) and low FM group (LFMG, GT_1_ + GT_2_). We separated the data from men and women for all statistical analyses. Two-tailed *p*-values were used for all analyses. A *p*-value <0.05 was considered to be statistically significant [18]. 

## 3. Results

### Demographic Data and Odds Ratios

The basic demographic data of the PRESENT study are shown in Table 1. Women had greater FM than men, unlike the result with skeletal muscle mass [18]. On univariate regression analysis, the overall OR of SI/WMC was 2.23 (95% confidence interval (CI), 1.34–3.71; *p* = 0.002) times higher in the third tertile (GT_3_) than in the first tertile (GT_1_). When men and women were separated, the OR of GT_3_ was 1.35 (95% CI, 0.62–2.94; *p* = 0.45) in men and 3.2 (95% CI, 1.60–6.40; *p* = 0.001) in women (Table 2). The findings were not statistically significant after adjusting for well-known risk factors for stroke such as age, hypertension, diabetes mellitus, dyslipidemia, and current smoking habits (Table 3). 

When subjects were divided into HFMG and LFMG, the HFMG showed an increased OR of SI/WMC only in women, but not in men (Table 2). Similar results were seen even after the well-known stroke risk factors (age, hypertension, diabetes mellitus, dyslipidemia, and current smoking habits) were adjusted (overall: OR, 1.38; 95% CI, 0.85–2.25, *p* = 0.198; men: OR, 0.93; 95% CI, 0.422–2.051; *p* = 0.86; women: OR, 2.02; 95% CI, 1.06–3.86; *p* = 0.03) (Table 3).

## 4. Discussion

The aim of the study was to understand the relationship between absolute fat mass (FM) and cerebral infarction. In general, the gold standard for the measurement of body FM is dual-energy X-ray absorptiometry (DEXA). However, DEXA involves using ionized radiation, which is hazardous to health, and an expensive device [18]. BIA, which is cheaper and less invasive, has been demonstrated to correlate well with DEXA [16].

Unlike BMI, which reflects both muscle and fat, abdominal obesity primarily reflects only fat. However, because body shape varies among races and individuals, it is difficult to define abdominal obesity accurately. The BIA method is easy, accurate, and safe, and can obtain absolute values for total FM. 

In order to assess the relationship between FM and cerebral infarction, we used SI/WMC as a dependent variable. The PRESENT project was a cross-sectional study performed on subjects who did not have a clinical cerebral infarction. We selected SI/WMC as the best indicator of cerebral infarction in patients without clinical infarction [26,27]. This was a both limitation of the study as well as an advantage. Since FM and muscle mass continuously change over time, SI/WMC detected using CT at the time of BIA measurement may be more meaningful than that during subsequent infarctions.

If fat cells are indeed responsible for cerebral infarction, then the increased accumulation of fat would in turn increase the frequency of cerebral infarctions. The PRESENT project suggests that higher values of total FM confer an increased risk of developing cerebral infarctions. However, only the data obtained from women showed a statistically significant association, even after adjustment for other risk factors.

There can be many reasons for the difference in results between men and women. We postulate that the difference in muscle mass between the two genders may be a cause. Men have greater muscle mass, relatively and absolutely, than women [28]. It is possible that the preventive effect of skeletal muscles on cerebral infarction counteracts the risk posed by fat. Muscles have shown marked stroke prevention effect in men (>50 years of age) [15,29,30]. A recent study showed that women had more central obesity than men, even with normal BMI [31]. We think that this may be a second reason for the difference in results between men and women in our study. Skeletal muscle mass decreases by up to 40% between 50–80 years, and we recruited subjects aged above 50 years for the current study [32]. Nevertheless, in the PRESENT project, men had greater muscle mass than women [18]. Therefore, it was assumed that the effect of fat was prominent only in women due to overall low muscle mass. If the results obtained were adjusted for muscle mass, the results for men would have been statistically significant.

If obesity, which is defined by BMI, is assumed to be a risk factor for cerebral infarction, then reducing weight would reduce the incidence of cerebral infarction. However, weight loss in elderly individuals has been shown to cause an increase in mortality rates [33]. This is thought to be due to a reduction in the protective muscle mass. Since BMI calculation is based only on height and weight, weight loss results in an improvement in BMI. 

We did not use an index that divided FM by other parameters such as height or weight. Height and weight are used in an algorithm for calculating FM by the InBody system. The exact algorithm used by the InBody system has not been disclosed for proprietary reasons. Therefore, dividing FM by height or weight would not have been appropriate for our analysis.

In previous reports, we argued that greater muscle mass correlates with a greater preventive effect on cerebral infarction [18]. Based on our previous reports and current research, it is likely that the concept of obesity based on BMI cannot be reliably applied to cerebral infarction risk assessment. Increased fat mass with age-related sarcopenia may increase the risk of cerebral infarction without changing the BMI. Therefore, when considering the risk of cerebral infarction, sarcopenic obesity (a lower muscle mass or muscle to fat ratio) is a more accurate indicator [34].

A cohort study would be the optimal observational study for such investigations. However, large-scale studies are difficult to carry out due to high costs and difficulties in management. We did not conduct a comprehensive survey but rather, extracted a representative sample. We randomly sampled subjects using a collective list of cohort members’ telephone numbers. Therefore, this study was of comparable consequence to a comprehensive survey. Moreover, this project (PRESENT) was carried out as part of a government-led health promotion project, and thus it was possible to receive government administrative aid.

Our basic hypothesis is that fat mass itself contributes to the risk of stroke. Here, we consider the absolute amount to be more important than the index. The use of BMI an indicator of relative fat mass was not used because it may lead to more confusing results.

Although meta-analysis has demonstrated that weight reduction reduces blood pressure, it is unknown whether weight reduction can directly reduce the risk of stroke. If fat reduction without muscle mass reduction can be detected using the BIA method, the effect of weight reduction on stroke risk reduction may be proven.

## 5. Conclusions

High FM may be an independent risk factor for ischemic stroke among stroke and dementia-free adults, especially in women.

## Figures and Tables

**Table 1:** Basic demographic data of the Prevention of Stroke and Dementia (PRESENT) study.

Variable	Normal (n = 613)	Silent Brain Infarction (SI)/White Matter Change (WMC) (n = 109)
Age (mean), years ± SD *	58.5 ± 7.1	68 ± 7.8
Men, n	276 (45%)	45 (41.3%)
Education(mean), years ± SD *	9.6 ± 4.4	7.5 ± 5.1
Hypertension, n *	307 (50.1%)	94 (86.2%)
Diabetes mellitus, n *	103 (16.8%)	34 (36.7%)
Hypercholesterolemia, n	199 (32.5%)	40 (36.7%)
Currently smoking, n	106 (17.3%)	19 (17.4%)
Fat mass, n	613 (100%)	109 (100%)
Tertile 1, n (%)	212 (34.6%)	26 (23.9%)
Tertile 2, n (%)	211 (34.4%)	31(28.4%)
Tertile 3, n (%)	190 (31.0%)	52 (47.7%)

* *p* < 0.05.

**Table 2:** Odds ratios of each tertile for silent infarction/white matter change (SI/WMC) on brain CT.

Variable	Reference	Odd Ratio	95% Confidence Interval	*p*
T2 (Men + Women)	T1	1.20	0.69–2.09	0.524
T3 (Men + Women)	T1	2.23	1.34–3.71	0.002
T2 (Men)	T1	1.19	0.54–2.63	0.666
T3 (Men)	T1	1.35	0.62–2.94	0.448
T2 (Women)	T1	1.21	0.55–2.62	0.629
T3 (Women)	T1	3.20	1.60–6.40	0.001
Total T3	T1 + T2	2.03	1.34–3.07	0.001
Men T3	T1 + T2	1.23	0.64–2.37	0.527
Women T3	T1 + T2	2.90	1.68–5.00	0.000

T1: first to 33rd percentiles; T2: 34th to 67th percentiles; T3: 68th to 100th percentiles.

**Table 3:** Odds ratios of each tertile for SI/WMC on brain CT after adjusting for well-known risk factors for stroke such as age, hypertension, diabetes mellitus, dyslipidemia, and current smoking habits.

Variable	Reference	Odd Ratio	95% Confidence Interval	*p*
Total T2	T1	1.20	0.69–2.09	0.524
Total T3	T1	2.23	1.34–3.71	0.02
Men T2	T1	1.31	0.49–3.48	0.585
Men T3	T1	1.08	0.41–2.84	0.874
Women T2	T1	1.24	0.50–3.05	0.631
Women T3	T1	2.28	0.99–5.25	0.051
Total T3	T1 + T2	1.34	0.85–2.25	0.198
Men T3	T1 + T2	0.93	0.422–2.051	0.86
Women T3	T1 + T2	2.02	1.06–3.86	0.03

T1: first to 33rd percentiles; T2: 34th to 67th percentiles; T3: 68th to 100th percentiles.
